# Enhancing Healing with Noncontact Low-Frequency Ultrasound in Fingertip Amputation Treatment: A Comparative Pilot Study

**DOI:** 10.1016/j.jhsg.2025.100843

**Published:** 2025-10-30

**Authors:** Robert W. Gomez, Abbigail Walsh, Kevin G. Valdes, Kristofer S. Matullo

**Affiliations:** ∗ Department of Orthopedic Surgery, St. Luke’s University Health Network, Bethlehem, PA; † Department of Occupational Therapy, Moravian University, Bethlehem, PA

**Keywords:** Amputation, Fingertip, Healing, Trauma, Ultrasound

## Abstract

**Purpose:**

Acute traumatic fingertip amputations are common injuries. This study aimed to evaluate the efficacy of noncontact low-frequency ultrasound (NCLF-US) as adjunctive therapy for treatment of fingertip amputations.

**Methods:**

A retrospective analysis was conducted on adult patients with an acute traumatic fingertip amputation without exposed bone between February 2022 and April 2023. Exclusions included vascular disease, active infection, surgical intervention, primary closure, or subsequent trauma. Patients received either NCLF-US therapy combined with local wound care or local wound care alone (LWCA). Data collected included age, sex assigned at birth, history of diabetes, and injury dimensions. Data were used to assess cohort demographics, injury characteristics, and clinical course. Nominal and continuous variables were analyzed using Fisher exact test and Student *t* test, respectively, with statistical significance set at *P* ≤ .05.

**Results:**

Among 19 digits, nine were treated with NCLF-US, and ten received LWCA. There was no considerable difference between cohorts in terms of age, sex, or history of diabetes. The NCLF-US cohort presented with an average wound size of 434.7 mm^2^ compared to the LWCA cohort at 123.0 mm^2^. Total time to healing in the NCLF-US cohort was 34.4 days compared to 49.2 days in the LWCA cohort. Healing rates for NCLF-US were 0.112 days/mm^2^ compared to LWCA at 1.038 days/mm^2^.

**Conclusions:**

The NCLF-US cohort exhibited larger initial fingertip amputations, while demonstrating a time to healing nine times faster than those treated solely with local wound care. These findings are encouraging and offer initial support for the consideration of NCLF-US as an adjunctive therapy for treatment of fingertip amputations.

**Type of study/level of evidence:**

Therapeutic IV.

Fingertips serve as the primary point of contact with our environment. Fingertip injuries rank among the most prevalent hand injuries, leading to nearly 5 million emergency department visits annually and commonly observed in populations composed of young men engaged in manual labor.^1^ Adults most commonly experience lacerations, crush injuries, and avulsions affecting the fingers. Although treatment of fingertip injuries is tailored to the individual patient, therapies aim to minimize pain, optimize healing, preserve sensory and motor function, and maintain an aesthetically acceptable appearance. Possible treatment options for fingertip amputations without exposure of the distal phalanx include primary closure, healing by secondary intention, completion/revision amputation, full or split thickness skin grafting, or flap coverage.^2^ However, when soft tissue allows, primary closure or healing by secondary intention have demonstrated positive and reliable outcomes.^3^ In a systemic review, Krauss and Lalonde^4^ reported conservative management of fingertip amputations had a mean time to healing of 4 weeks, quicker return to work, satisfactory aesthetic appearance, and comparable two-point discrimination to the uninjured hand.

Contemporary technologies, such as noncontact low-frequency ultrasound (NCLF-US) have demonstrated the potential for improved healing of chronic wounds. Maan et al^5^ demonstrated NCLF-US therapy improved neovascularization and wound closure in diabetic mice following a full-thickness wound. Increased expression of neovascularization markers including vascular endothelial growth factor, stromal cell-derived factor 1, and CD31 were observed in mice treated with NCLF-US versus standard of care.^5^^,^^6^ Other studies have demonstrated the efficacious use of NCLF-US in nonhealing chronic wounds compared with standard of care through a reduction in wound size and proinflammatory cytokines ultimately allowing for considerably improved tissue regeneration.^6^, ^7^, ^8^, ^9^, ^10^, ^11^, ^12^, ^13^, ^14^ However, the literature on NCLF-US has not examined its efficacy in the treatment of acute hand injuries.

Given the significance of optimized healing seen in chronic wounds, we hypothesized that the use of NCLF-US would be associated with expedited healing of fingertip amputations without exposed bone through secondary intention. This study aimed to compare the efficacy of NCLF-US in combination with standard treatment for secondary healing versus the standard treatment alone.

## Materials and Methods

This retrospective, case-controlled study received approval from our institutional review board. We analyzed consecutive patients treated for acute traumatic fingertip amputations without exposed bone, conservatively, between February 2022 and April 2023 using an electronic medical record. All patients received care within a single regional health network, accessing initial treatment through either consultation through the emergency department or the treating surgeon’s outpatient clinic. All care was provided by a single fellowship-trained orthopedic hand surgeon and a certified, licensed hand therapist.

Inclusion criteria were individuals aged ≥18 years with an acute traumatic fingertip amputation without exposed bone or tendons (Allen 1, Tamai level 1; International Classification of Diseases codes S68.5 and S68.6). Exclusion criteria included patients <18 years old, injuries involving exposed bone, presentation >72 hours from the time of injury, those with diagnosis of vascular disease, patients prescribed antibiotics prior to the initiation of treatment, individuals with clinical suspicion or diagnosis of infection, and/or those unable to comply with prescribed therapy sessions. Additionally, patients who underwent primary closure or surgical intervention or experienced subsequent trauma following treatment initiation were excluded.

For eligible patients, treatment consisted of either adjunctive NCLF-US therapy in combination with local wound care treatment within our health network or local wound care alone (LWCA). Patients in both groups were instructed in the Lalonde protocol for local wound care. The Lalonde protocol involves rinsing the site of injury with clean water daily, applying a grease layer, such as Vaseline, with a 1 inch gauze wrap, and securing it with clean Coban tape.^15^ Given the retrospective nature of our study, achieving an even distribution of patients within these groups is challenging. Patients were placed in the NCLF-US and LWCA group based on the patient’s ability to attend NCLF-US sessions, patient preference, and financial factors.

During each office visit, patients received thorough instructions and demonstrations for appropriate care using the Lalonde protocol, with opportunities to ask questions and receive clarification. Comprehension and understanding were assessed using the teach-back method to promote compliance. Patients were instructed to adhere to the protocol daily from the initial evaluation with the hand surgeon until full healing, defined as complete re-epithelialization of the epidermal layer, occurred. All necessary supplies were provided, and the importance of maintaining clean bandages were repeatedly emphasized.

For patients in the NCLF-US cohort, all sessions were conducted using a single NCLF-US device (UltraMIST, Sanuwave Wound Care Technologies, MN) following appropriate manufacturer guidelines. Sessions occurred one to three times a week, depending on patient insurance coverage, to avoid creating unnecessary financial burden for the patient. Each NCLF-US session was standardized at our institution to five minutes. NCLF-US therapy involves delivering low-frequency (40 kHz), low-intensity (0.1-0.8 W/cm^2^) ultrasound energy via sterile saline mist.^11^ Each patient was supplied with a labeled sterile saline bottle, securely stored in a locked cabinet between sessions. The head of the NCLF-US device was changed after each use to limit contamination, in accordance with manufacturing requirements.

Data collection included patient demographics (age, sex, and diagnosis of diabetes mellitus) and measurements used to characterize the initial fingertip amputation injury and to monitor the clinical course of healing. For the follow-up protocol, patients were seen every week by either the surgeon or the physician assistant depending on patient and office scheduling availability. Standardized measurements were taken at initial enrollment and during each subsequent follow-up until full healing. Measurements were also collected during therapy sessions for patient in the NCLF-US group. Measurements included length and width, determined by measuring the greatest distance on orthogonal axes to calculate surface area in millimeters squared. Measurements were taken to the nearest millimeter using a ruler. All standardized measurements were performed by a single unblinded hand therapist to minimize variability.

Patient demographics and fingertip amputation characteristics were compiled for cohort analysis. Categorical and continuous variables were analyzed using Fisher exact test and Student *t* test, respectively. Statistical significance was determined with a significant level set at *P* ≤ .05.

## Results

Eighteen patients met the inclusion criteria for enrollment, resulting in the treatment of 19 digits. Among the 19 fingertip amputations treated, nine were in the NCLF-US cohort, and 10 were in the LWCA cohort. One individual presented with injuries to two digits and was assigned to the NCLF-US cohort. All patients attended prescribed appointments without missing or rescheduling consecutive appointments, and were followed until their injuries were fully healed. Furthermore, all patients completed their prescribed therapy with no crossover between groups.

Patient demographics and clinical experiences were similar between the cohorts in terms of age, sex, history of diabetes, and total number of visits (Table 1). The overall average age was 51.6 years, with 15 men and 4 women. Three patients previously diagnosed with diabetes. In the NCLF-US cohort, the average age was 59.9 ± 19.8 years. The cohort consisted of eight men, one woman, and one patient having a history of diabetes. Similarly, in the LWCA cohort, the average age was 44.2 ± 13.7 years. The cohort consisted of seven men, three womem, and two patients with diabetes. The average total number of visits was 8.9, with NCLF-US having a slightly higher average total number of visits (11 vs 7), although this difference was not significant (*P* = .107).

**Table 1:** Patient Demographics and Clinical Characteristics

Patient Demographics	NCLF-US + Local Wound Care	Local Wound Care Alone	Total	*P* Value
No. of digits	9	10	19	
Age, mean (SD)	59.9 (19.8)	44.2 (13.7)	51.6 (18.2)	.067
Sex				
Male	8 (89%)	7 (70%)	15 (79%)	.582
Female	1 (11%)	3 (30%)	4 (21%)	
History of diabetes				
Yes	1 (11%)	2 (20%)	3 (16%)	>.999
No	8 (89%)	8 (80%)	16 (84%)	
Total no. of visits, mean (SD)	11.0 (7.0)	7.0 (2.5)	8.9 (5.4)	.107

The NCLF-US cohort exhibited larger initial fingertip amputations but healed in fewer total days and at a faster rate compared to patients who were treated with LWCA. On average, the NCLF-US cohort experienced significantly larger initial fingertip amputations, with an average area of 434.7 ± 273.6 mm^2^, compared with 123.0 ± 150.3 mm^2^ in the LWCA cohort (*P* = .006). Additionally, the NCLF-US cohort demonstrated a shorter time to healing (34.4 ± 12.6 vs 49.2 ± 17.6 days, *P* = .053), by healing at a rate approximately nine times faster than the LWCA cohort (0.112 ± 0.091 vs 1.038 ± 0.812 days/mm^2^, *P* = .003), as illustrated in the Table 2.

**Table 2:** Healing Characteristics

Healing Characteristics	NCLF-US + Local Wound Care	LWCA	*P* Value
Length (mm), mean (SD)	22.2 (7.4)	17.6 (6.8)	.176
Width (mm), mean (SD)	18.2 (7.1)	6.1 (5.4)	<.001
Area (mm^2^), mean (SD)	434.7 (273.6)	123.0 (150.3)	.006
Time to healing (d), mean (SD)	34.4 (12.6)	49.2 (17.6)	.053
Healing rate (d/mm^2^), mean (SD)	0.112 (0.091)	1.038 (0.812)	.003

## Discussion

Fingertip injuries are common yet diverse, requiring individualized treatment to minimize pain, complications, and convalescence while optimizing function.^16^ Although prior research supports the use of NCLF-US in chronic wounds, such as venous leg or diabetic foot ulcers, its effectiveness in acute injuries, particularly fingertip amputations, is absent.^17^ Our study aimed to address this gap in knowledge by evaluating the efficacy of NCLF-US in the treatment of acute traumatic fingertip amputations without exposed bone and allowing for healing by secondary intention.

Although the mechanism of action of NCLF-US in acute injuries is not well defined, studies in chronic wounds provide some insight. Escandon et al^13^ evaluated biopsies before and after NCLF-US therapy in venous leg ulcers refractory to care. All patients showed a decrease in wound size and considerable reduction in tumor necrosis factor-alpha and interleukin-1 levels after four weeks of treatment, three times a week. The authors suggested that this decrease in proinflammatory cytokines likely played a role in allowing the wounds to transition from chronic to acute, via creating a local environment more conducive to healing. However, similar to our study, the limited sample size prevents the ability to make strong conclusions.

The most similar studies would be from Honaker et al,^18^^,^^19^ who retrospectively reviewed the efficacy of NCLF-US in the treatment of acute injuries and reported their findings in two separate studies. In both studies, they evaluated the efficacy of NCLF-US in the care of admitted patients with newly diagnosed deep tissue pressure injuries, finding that NCLF-US resulted in a greater decrease in wound area and severity compared to standard of care alone. However, it is essential to acknowledge the significance of the differing etiologies between fingertip amputations and deep tissue injuries.

Our findings support the hypothesis that NCLF-US is associated with expedited healing of fingertip amputations. Patients in the NCLF-US cohort demonstrated larger initial injuries but achieved complete healing quicker (34 vs 49 days) and healed at increased rate per mm^2^ (0.112 vs 1.038 days/mm^2^) compared to those treated with local wound care using a Lalonde protocol alone. These results suggest adjunctive NCLF-US has potential for supporting fingertip amputation treatment goals by optimizing healing in this patient population.

Despite our many strengths to this study, including its novelty and standardized treatment protocol, several limitations should be acknowledged. The retrospective design and small sample size limit external validity and is associated with the inherent limitations of retrospective comparisons. This was an unadjusted comparison, and our treatment groups were not large enough for an appropriate regression analysis. Thus, confounders are likely present and should be assessed. Additionally, because this was an unblinded study, potential selection and observer bias should be acknowledged. Furthermore, a larger sample size would allow for further patient stratification based on factors such as the most recent HbA1c, nutritional status, smoking or nicotine use history, which are known to impact healing. Additionally, although patient education was standardized and robust, it is unclear what percentage of patients appropriately followed the Lalonde protocol and how this may have influenced results. Patients in the NCLF-US group were seen more frequently for therapy sessions, which potentially influenced the degree of compliance to the local wound care protocol. Future research addressing these limitations through single blinded, prospective, randomized controlled trials with a larger patient population for further evaluation of the benefits of NCLF-US in this patient population is ongoing. Nonetheless, the results regarding optimization of healing and decreased patient morbidity are promising.

In conclusion, our study provides valuable insights into the efficacy of NCLF-US as adjunctive therapy for acute traumatic fingertip amputations without exposed bone. Although further research is needed to fully understand its mechanism of action and confirm its efficacy, our findings support consideration of its use in optimizing healing outcomes in this patient population.

## Conflicts of Interest

No benefits in any form have been received or will be received related directly to this article.
